# Surface Body Temperature of Full-Term Healthy Newborns Immediately after Birth—Pilot Study

**DOI:** 10.3390/ijerph16081312

**Published:** 2019-04-12

**Authors:** Anna Lubkowska, Sławomir Szymański, Monika Chudecka

**Affiliations:** 1 Department of Functional Diagnostics and Physical Medicine, Faculty of Health Sciences Pomeranian Medical University in Szczecin, ul. Żołnierska 54, 71-210 Szczecin, Poland; anna.lubkowska@pum.edu.pl; 2 Department of Obstetrics and Pathology of Pregnancy, Pomeranian Medical University in Szczecin, ul. Żołnierska 48, 71-210 Szczecin, Poland; slawomir.szymanski@pum.edu.pl; 3 Department of Functional Anatomy and Biometry, Faculty of Physical Education and Health Promotion, University of Szczecin, al. Piastów 40b/6, 71-065 Szczecin, Poland

**Keywords:** newborns, brown tissue, thermoregulation, infrared camera

## Abstract

The aim of the study was to perform an evaluation of chosen body surface temperatures in neonates immediately after birth, and to seek a relationship between those temperatures and the factors related both to the mother and newborn. The study included 74 healthy newborns. Maternal age, body weight, body mass index before pregnancy and on delivery day, birth and pregnancy order, newborn sex, birth weight, body length, pregnancy week on delivery, as well as newborn gasometric test results were collected. The highest temperature values were observed in the chest of the newborn. Significant relationships between the temperature of the evaluated areas were found. The parameters that correlated positively with the temperature of the back region were maternal body weight (both before pregnancy and on delivery day) as well as weight gain during pregnancy. The core and surface temperatures of the body are one of the most important elements of neonatal homeostasis and any changes constitute a risk to the newborn’s health. It seemed that according to the surface temperature, the most important area that must be evaluated is the neonate’s back, as it is most affected by appropriate weight gain during pregnancy.

## 1. Introduction

The maintenance of a constant body temperature is important to all humans, but even more so for newborn babies (neonates)—it is one of the major tasks of extrauterine adaptation [1,2,3,4,5].

Foetal body temperature depends on the temperature of the mother’s blood, and due to increased metabolic processes in an unborn baby it is slightly higher than the temperature of her body, at about 37 °C. Newborn infants lose heat rapidly at birth and during the first half hour of life (about 2 °C just several seconds after birth). The reason for the sudden cooling of the newborn body is the delivery room temperature, which is lower than the mother’s temperature, and the evaporation of amniotic fluid from their skin [6]. Full-term newborns are warm-blooded, while those born prematurely have no such ability due to the late-maturing thermoregulation system (last trimester of pregnancy). The energy used by humans to generate heat comes from three basic sources—basal metabolism, shivering thermogenesis and non-shivering thermogenesis. Newborns use only the last of the available mechanisms, and the source of generated energy is brown adipose tissue (BAT) which is deposited from 26–28 weeks of foetal life and constitutes about 10% of the full-term newborn body weight. BAT is highly specialised, well-vascularised, and is located mainly between the shoulder blades, in the armpits, in the mediastinum, along the spine, and around kidneys and adrenal glands. Exposure of a newborn to cold also increases the action of noradrenaline which stimulates BAT breakdown and generation of a large amount of heat. However, its reserves are non-renewable [7]. The reasons for the greater susceptibility of the newborn body to cooling are considered to be their high ratio of body surface area to weight; high heat loss by evaporation, which is associated with skin immaturity; deficiency of subcutaneous adipose tissue acting as an insulator; poorly developed muscles; as well as low ability to regulate blood flow through the skin. In addition, the immaturity of the circulatory and respiratory systems is also a factor that impedes the process of proper temperature regulation. Newborns, particularly preterm and low birth weight (LBW) infants, have limited capacity for thermoregulation during the first weeks of life.

There is no consistent definition of a normal newborn body temperature, and consequently data that would allow for pooled risk estimates for newborn hypothermia are still incomplete. Temperatures have been shown to vary widely in healthy newborns, and standard medical textbooks disagree on the lower normal limit, ranging from 35.5 to 36.5 °C, as well as the normal upper level, citing values from 37.0 to 37.9 °C [8,9]. To assess the core and axillary temperature of newborns, a rectal digital thermometer is used as the standard method. Mercury-in-glass, gallium-in-glass, digital, analogue electronic, chemical and infrared thermometers are all accurate options. Most developed institutions use infrared tympanic thermometers, which are a quick and accurate method to measure newborn body temperature [10].

In the available literature, there are very few studies related to the thermal imaging of body surface temperature in newborns, and reports on body surface temperature distribution in newborn infants both immediately after birth and in the subsequent days of life, which may prove to be a useful method in assessing the risk of hypothermia, one of the symptoms of which is cold skin in newborns. There are also no analyses regarding the effect of factors related to the mother’s body, such as maternal age, body weight change during pregnancy and birth order, and newborn traits—including birth weight, sex, pregnancy week on delivery, and biochemical parameters—on the distribution of these temperatures.

Therefore, the aim of this study was to analyse the values and distribution of the temperatures of selected body surfaces in full-term healthy newborns immediately after birth using a thermal imaging camera, and to seek a relationship between these temperatures and the factors related to both the mother and the newborn. The following factors were included in the analysis: maternal parameters, including age, body height, body weight and body mass index (BMI) before pregnancy and on delivery day, birth order and pregnancy order, and neonatal parameters, including birth weight, body length, pregnancy week on delivery, and blood gasometry results (pH, pO_2_, HCO_3_, ctCO_2_, sO_2_).

## 2. Material and Methods

The initial study included a total of 100 newborns born January–July 2018 in the Gynaecology and Obstetrics Ward, SPZOZ Hospital (an independent public healthcare institution) in Choszczno. The study was approved by the Bioethics Committee of the Pomeranian Medical University in Szczecin (KB 0012-15/12).

Immediately after birth, in the first minute after birth, the selected body surface temperatures were measured, without wiping the newborn, before further activities such as placing them in an open radiant warmer, and before the setting of neonatal clinical care. The newborn was supported by the obstetrician under the arms in a vertical position and the temperature was recorded on the anterior surface, in the chest region (the area below the clavicular line to the costal arch line); on the posterior surface, in the upper back region (the area to the line connecting the inferior angles of both scapulae); and in the newborn’s forehead region.

Ultimately, the analysis only included the thermograms of those newborns who scored 9–10 points on the Apgar scale (from the first minute) and were assessed as healthy by the neonatologist in the subsequent evaluation. An additional gradient for testing was a medical qualification confirming the newborns’ condition and the healthy status of the mother. Healthy status was considered to include women with a normal or overweight pre-pregnancy BMI (18.5–29.0), who experienced normative weight gain during their pregnancy, in the 11.5–16 kg range for women with a normal pre-pregnancy weight and 7–11.5 kg for overweight women [11]. All examined women had a natural childbirth.

Finally, 74 newborn thermographs were included in the partial analysis. The mean surface temperature (T_mean_) was calculated for each selected area, as we found it to be more representative for a given body area than minima and maxima.

Thermal images were taken with a thermal imaging camera (FLIR E60) in the delivery room, with all necessary precautions and without affecting the course of labour.

The tests were carried out following the standards of the European Thermographic Association [12]. During imaging, the room temperature and humidity were almost constant at the measurement site, at appropriately 26 °C and 55–60%. For each use, the camera was positioned in a straight line to the subject, at a distance of 1.5 m. Skin emissivity was adopted as 0.98. The FLIR Tools software was used to evaluate the thermograms.

Additionally, data on maternal age, body height, body weight and BMI before pregnancy and on delivery day, birth and pregnancy order, as well as newborn sex, birth weight, body length, pregnancy week on delivery, and newborn gasometric tests results (pH, pO_2_, HCO_3_, ctCO_2_, sO_2_) were collected. 

### Statistical Analysis

Calculations were performed with the use of the STATISTICA 10 software (StatSoft Company, Krakow, Poland). The temperature in the analysed areas was normally distributed (which was verified with the Shapiro–Wilk test). We presented the results of the measurements as arithmetic means and their standard deviations. The mean temperatures (T_mean_) of selected newborn body areas, including back, chest and forehead, were determined and then compared with the Student’s t-test to verify whether the differences between them were statistically significant.

The Spearman test was performed for the relationship analysis between T_mean_ values, as well as to verify whether the chosen maternal traits (age, pregnancy order, birth order, body height, body weight before pregnancy and on delivery day, body weight gain during pregnancy, BMI before pregnancy and on delivery day) and newborn traits (sex, body length and birth weight, pregnancy week on delivery, and gasometric tests (pH, pO_2_, HCO_3_, ctCO_2_, sO_2_) had a significant effect on the temperature of the selected body surfaces. In addition, the relationship between the examined characteristics of the mothers and their newborns was assessed.

## 3. Results

Descriptive statistics of the values of the maternal and neonatal traits under analysis are presented in Table 1.

Descriptive statistics of the T_mean_ temperature values of newborns’ body areas are presented in Table 2. In all examined newborns, the range of surface temperature of the areas under analysis was within the wide limits of 33.2 to 37.2 °C. Between the analysed areas, statistically significant differences in mean temperature values occurred. The lowest mean temperature (T_mean_) values were recorded in the newborns’ forehead region (M = 35.05 °C), while the highest values were recorded in the newborns’ chest region (M = 35.6 °C).

Importantly, the results of Spearman’s rank-order correlation analysis showed significant relationships between the T_mean_ values of the areas under analysis (Table 3), with the recorded temperature values in all body areas of the examined newborns being dependent on each other.

Another goal of the conducted research was to search for a relationship between maternal traits—such as age, blood group, pregnancy order, birth order, body height, body weight before pregnancy and on delivery day, body weight gain during pregnancy, BMI before pregnancy and on delivery day—and neonatal traits—including—sex, body length and birth weight, week of the prenatal period at birth, blood gas test results (pH, pO_2_, HCO_3_, ctCO_2_, sO_2_), and T_mean_ on newborn back, chest and forehead surfaces.

Analysis of the results of Spearman’s rank-order correlation showed that virtually all of the analysed traits, both on the part of the mother and the newborn, did not affect the body surface temperatures. Interestingly, the parameters that correlated positively, but only with the surface temperature of the back region, were maternal body weight, both before pregnancy (*p* < 0.05; r = 0.311) and on delivery day (*p* < 0.05; r = 0.383), and the increase in body weight during the pregnancy (*p* < 0.05; r = 0.35).

When assessing the relationship between the studied characteristics of mothers and their newborns, a positive relationship was found between the pregnancy week and birth weight (*p* < 0.05, r = 0.24) and the newborn’s body length (*p* < 0.05, r = 0.27).

## 4. Discussion

Adapting to the cold extrauterine environment after birth is a great challenge for the newborn. Due to their high surface-area-to-volume ratio, infants tend to lose more heat to the environment as compared to adults [13]. The imperfection of the thermoregulation system in newborns, results among other reasons from the immaturity of the circulatory and respiratory systems, their very thin skin, and the low content of subcutaneous fat, which directly affect the ineffective body thermal balance, including the very fast loss of endogenous heat (e.g., due to evaporation). Heat loss in newborns occurs in several ways. The energy loss is substantial immediately at delivery, when the environmental temperature surrounding the baby drops from 37 °C in the maternal womb to the usually cooler air temperature, evaporative heat loss begins at a rate of 0.58 kcal·mL^−1^ of fluid evaporated. Evaporation often occurs with amniotic fluids during the first minutes of life [14]. In the presented studies, an attempt was made to estimate the body surface temperature of healthy, full-term infants immediately after birth.

Based on the guidelines for thermographic tests, three different areas of newborn body surface (where is possible to register temperature correctly) were selected for the analisys. Due to the BAT distribution typical for newborns, the upper back region was selected for analysis. The next analysed areas were the chest and forehead areas, as they are generally considered significant for heat exchange. Conditions that were taken into account in order to regard the analysed temperatures as normative included physiological pregnancy, healthy status of the women, and appropriate weight gain during the pregnancy. Weight gain in pregnancy is one of the anthropometric factors affecting the condition of the newborn and is related to the lowest risk for pregnancy–delivery complications [15].

In the studied population of newborns, the highest T_mean_ was recorded on the chest surface, as a result of the function of internal organs and endogenous heat transmission generated in this area. At the same time, small subcutaneous fat layers cannot act as an insulator, which facilitates heat loss. The temperature of the back surface in newborns was comparable to the chest temperature, which may be directly affected by brown adipose tissue (BAT) distribution in this region in newborns. The function of brown adipocytes is critically related to uncoupling protein 1 (UCP1), a mitochondrial protein uniquely expressed in this cell type [16]. BAT thermogenesis is principally dependent on the β-adrenergic-mediated activation of lipolysis and the subsequent degradation of fatty acids via UCP1, which uncouples mitochondrial oxidative phosphorylation to dissipate the electrochemical gradient as heat instead of ATP synthesis. This β-adrenoceptor–UCP1 system has been recognised as an intriguing target for the control of body energy balance [17]. The areas predominantly containing brown adipocytes had a greater density of capillaries and noradrenergic fibres than those predominantly containing white adipocytes. Our previous research allowed us to develop thermal maps in adult women and men, with which the results obtained in this study were compared. The obtained chest and back temperature values were much higher in newborns (on average by 2 °C) than the temperature of respective areas in young healthy women (33.47 °C and 33.55 °C, respectively) and men (33.15 °C and 33.92 °C, respectively) [18]. Most likely, an overall undifferentiated pattern of skin temperature immediately after birth is associated with the nature of foetal circulation. The presence of the ductus arteriosus in prenatal life allows for the inclusion of the right ventricle output into systemic circulation instead of the lungs being respiratory inactive in this period of life. At birth, there is a sudden change in haemodynamic conditions. With the first breath and cutting of the umbilical cord, there is a decrease in pulmonary vascular resistance and an increase in systemic vascular resistance, which changes the flow direction, and the previous (intrauterine) blood shunting from the pulmonary artery to the aorta is reversed left-to-right. Changes in the composition of the blood gases flowing through the ductus and reduction in prostaglandin E1 and E2 blood concentrations usually induces its constriction and then closure, leaving the arterial ligament. Blood oxygenation takes place in the lungs, not in the placenta, and peripheral vascular resistance becomes higher and the core of the body becomes warmer than its peripheral parts [19]. The data available in the literature relating to the values obtained from non-contact forehead infrared temperature measurements are only punctual in nature, whereas our results are more representative because they referred to the entire forehead area.

The next step of the conducted study was to search for factors from both the mother and the newborn that could determine the temperature values of the analysed newborn areas. It was expected that such a factor could be the newborn birth weight and body length, as their high ratio of body surface to weight induces a large loss of heat by evaporation. However, such relationships were not found. Another hypothesised potentially significant factor determining the temperature variation was the sex of the newborn. It is well-known that full-term and preterm female infants have a more centralised pattern of subcutaneous adipose tissue than male infants, as well as a higher amount of subcutaneous body fat [20]. However, no relationships were found in this case, either. Only the mother’s body weight, both before pregnancy and on delivery day, as well as the increase in body weight during the pregnancy correlated positively with the temperature of newborn back surface. When the pregnancy proceeded correctly in terms of the recommended weight gain for women, this was consistent with the higher fat mass content in infants born from well-fed mothers. This perhaps determines the increased content of both yellow and brown adipose tissue, and consequently determines the appropriate range of non-shivering thermogenesis, which is the most important mechanism protecting the newborn body temperature from cooling. It has been documented that heat production in infants depends on the amount of brown fat and the levels of 5’/3’-monodeiodinase and thermogenin that occur only later in foetal development [21]. At the same time, the limited content of subcutaneous adipose tissue does not constitute protection against endogenous heat loss, which affects the temperature of the newborn’s skin. Both a decreased and an increased core temperature increases the metabolic rate of newborns, who have only a very limited ability to maintain a normal temperature and easily become hypothermic or hyperthermic, but hypothermia seems to carry a higher risk of complications [22]. Newborns are unable to maintain their body temperature on their own without thermal protection, especially when placed in a colder environment, as their core temperature decreases at a rate of 0.2 °C to 1.0 °C per minute and finally may lead to death from the cessation of metabolic activities [14]. The WHO recognises maintaining an appropriate body temperature as a primary principle of newborn care and recommends thermal protection for all infants, with special attention for infants who are premature or small for their gestational age (for example, <2.5 kg at birth or born before 37 weeks gestation), and also recommends frequent measurements (from every hour in a seriously ill baby, to two to four times per day in a small or very small baby, and once daily in an infant progressing well) [23].

In the literature, there is little information on the results of research into the assessment of thermoregulation mechanisms in newborns or even the assessment of newborn body temperature and the factors that modify it. Pioneering research in this area used skin or rectal thermistors to assess changes in body temperature. Very early studies on newborn temperature regulation showed that 15 min after birth the newborn skin temperature fell almost by 4 °C, after the first hour of life the temperature finally levelled at approximately 33 °C, and the rectal temperature reached approximately 35°C after 2 h at approximately 25 °C of room temperature. From the age of 15 min, there was a temperature gradient of 2.5 to 3 °C between the skin and the rectum. Moreover, shivering does not occur in the first 15 min—it is known that shivering is not regularly involved in a newborn’s response to cold stress [24].

Currently, the use of the thermographic method provides the opportunity to perform quick and non-invasive measurements of body surface temperature and to evaluate its distribution and potential changes, including in newborns immediately after birth, although such studies are scarce. According to the literature data about the temperature distribution in full-term newborns, immediately after birth there is a slight thermal variation across the entire body surface, and the skin temperature is much lower than the core temperature. In this study, it was again demonstrated that during the first hour of life the mean temperatures of selected newborn body surfaces began to decrease in relation to their core temperature. This study showed that the use of thermal imaging for assessing the body surface temperature can provide valuable information on changes in body temperature and perfusion resulting from changes in blood flow.

## 5. Conclusions

In conclusion, thermal imaging is a sensitive and non-invasive method that, based on the analysis of thermographically defined areas, allows the determination of minimum, maximum and mean temperatures, providing more reliable information than the point reading from infrared laser thermometers. Although the highest body surface temperature values in the newborns occurred on the chest surface, the temperature of the back was positively correlated with the weight gain of women retaining their nutritional regimen during pregnancy, which is a determinant of coping with thermal stress by the newborn.

The core and the surface temperature of the body is one on the most important elements of neonatal homeostasis and any changes in it constitute a risk to the newborn’s health and life. On the other hand, temperature is a reflection of many changes in the newborn’s body. Therefore, the precise, quick and non-invasive monitoring of the temperature of different body areas is extremely important. While the literature provides the ranges of normal newborn core body temperatures, the acceptable limits of surface temperature values are still not established. This was made possible by the use of thermography in our study, and the temperature values that we obtained are an important contribution to the research into the thermal mapping of healthy, full-term newborns.

## Figures and Tables

**Table 1:** Descriptive statistics of the values of maternal and neonatal traits under analysis.

N = 74	Mean	Standard Deviation	Min	Max
maternal age (years)	27.0	5.66	18.0	41.0
pregnancy order	1.8	1.03	1	5
birth order	1.5	0.75	1	4
pregnancy week	38.5	2.59	37.0	41.0
body height (cm)	165.8	5.38	150	176
body weight before pregnancy (kg)	63.1	8.22	45	87
body weight on delivery day (kg)	76.2	7.98	57.5	98.5
weight gain (kg)	13.1	2.16	7	16
BMI before pregnancy (kg/m^2^)	22.9	2.74	18.21	29.4
BMI on delivery (kg/m^2^)	28.4	4.42	22.14	47.46
birth weight (g)	3430	523.24	2300	4960
body length (cm)	55.7	3.38	47.0	64.0
pH	7.36	0.07	7.19	7.49
pCO_2_ (mmHg)	40.67	7.36	29.20	64.50
HCO_3_ (mmol/L)	22.16	2.14	16.60	26.10
pO_2_ (mmHg)	30.03	6.83	17.0	44.0
ctCO_2_ (mmol)	17.61	5.76	5.0	40.90
sO_2_ (%)	51.17	16.72	17.00	82.50

**Table 2:** Descriptive statistics of the T_mean_ temperature values of newborns’ body areas under analysis.

Mean		Standard Deviation	Min	Max	Student’s *t*-Test
T_mean_ back (°C)	35.51	0.72	34.00	37	NS
T_mean_ chest (°C)	35.64	0.68	34.30	37.2	T_mean_ forehead ***
T_mean_ forehead (°C)	35.05	0.74	33.2	37	T_mean_ back ***

*** significance level of *p* < 0.001.

**Table 3:** The results of Spearman’s rank-order correlation analysis of the T_mean_ temperatures under analysis.

Variable	Spearman’s Rank-Order Correlation with Significance Level of *p* < 0.05		
	T_mean_ Back	T_mean_ Chest	T_mean_ Forehead
T_mean_ back		0.742 *	0.630 *
T_mean_ chest	0.742 *		0.654 *
T_mean_ forehead	0.630 *	0.654 *	

* significance level of *p* < 0.05.
